# Effects of Heparin and Enoxaparin on APP Processing and Aβ Production in Primary Cortical Neurons from Tg2576 Mice

**DOI:** 10.1371/journal.pone.0023007

**Published:** 2011-07-29

**Authors:** Hao Cui, Amos C. Hung, David W. Klaver, Toshiharu Suzuki, Craig Freeman, Christian Narkowicz, Glenn A. Jacobson, David H. Small

**Affiliations:** 1 Menzies Research Institute Tasmania, University of Tasmania, Hobart, Tasmania, Australia; 2 School of Pharmacy, University of Tasmania, Hobart, Tasmania, Australia; 3 Laboratory of Neuroscience, Graduate School of Pharmaceutical Sciences, Hokkaido University, Kita-ku, Sapporo, Japan; 4 Department of Biochemistry and Molecular Biology, Monash University, Clayton, Victoria, Australia; 5 Division of Immunology and Genetics, The John Curtin School of Medical Research, Australian National University, Canberra, Australia; Federal University of Rio de Janeiro, Brazil

## Abstract

**Background:**

Alzheimer's disease (AD) is caused by accumulation of Aβ, which is produced through sequential cleavage of β-amyloid precursor protein (APP) by the β-site APP cleaving enzyme (BACE1) and γ-secretase. Enoxaparin, a low molecular weight form of the glycosaminoglycan (GAG) heparin, has been reported to lower Aβ plaque deposition and improve cognitive function in AD transgenic mice.

**Methodology/Principal Findings:**

We examined whether heparin and enoxaparin influence APP processing and inhibit Aβ production in primary cortical cell cultures. Heparin and enoxaparin were incubated with primary cortical cells derived from Tg2576 mice, and the level of APP and proteolytic products of APP (sAPPα, C99, C83 and Aβ) was measured by western blotting. Treatment of the cells with heparin or enoxaparin had no significant effect on the level of total APP. However, both GAGs decreased the level of C99 and C83, and inhibited sAPPα and Aβ secretion. Heparin also decreased the level of β-secretase (BACE1) and α-secretase (ADAM10). In contrast, heparin had no effect on the level of ADAM17.

**Conclusions/Significance:**

The data indicate that heparin and enoxaparin decrease APP processing via both α- and β-secretase pathways. The possibility that GAGs may be beneficial for the treatment of AD needs further study.

## Introduction

Alzheimer's disease (AD) is the most common neurodegenerative disease in the elderly and is a major cause of dementia [1], [2]. AD is characterized by the deposition of amyloid plaques in the brain [3], [4], the major component of which is the β-amyloid protein (Aβ), a 40–42 amino-acid residue polypeptide [5], [6] that is generated from the β-amyloid precursor protein (APP) [7] by the β-site APP cleaving enzyme-1 (BACE1) [8], [9], [10], [11] and γ-secretase [12]. Cleavage of APP by BACE1 yields a C-terminally truncated fragment (C99) which is subsequently cleaved by γ-secretase to yield at least two Aβ species, the major product Aβ40, which contains 40-amino-acid residues and Aβ42, which contains an extra two amino-acid residues at its C-terminus. APP can also be cleaved by α-secretase within the Aβ sequence [13], [14] to form sAPPα and C83, which thus precludes formation of Aβ [15]. Several members of the disintegrin and metalloprotease (ADAM) family have been proposed as α-secretases although ADAM10 is likely to be the most important contributor to this activity [16], [17], [18], [19]. Oligomeric forms of Aβ are now thought to be the major toxic species [20], [21], [22], [23]. Therefore, therapeutically targeting the production, aggregation, clearance or neurotoxicity of Aβ is a central theme of AD research [24].

A number of studies indicate that glycosaminoglycan (GAG) may have value for the therapeutic treatment of AD. GAGs are linear polymers consisting of repeated disaccharide units. Heparin is a typical GAG and has a highly sulfated structure. A low molecular weight (LMW) derivative of heparin, enoxaparin, is generated by alkaline depolymerization of heparin benzyl ester. Both heparin and LMW heparins have been widely used as anticoagulant and antithrombotic drugs [25], [26]. LMW heparins are now generally regarded as safer and more effective for the treatment of cardiovascular problems than unfractionated heparin [27].

LMW GAGs may also be suitable agents for the treatment of brain diseases as they can cross the blood-brain barrier [28], [29]. GAGs can inhibit Aβ toxicity [30], [31], [32], [33] and may have neuroprotective effects [34], [35]. In addition, peripheral administration of enoxaparin has been reported to reduce Aβ load [32] and improve cognition in APP transgenic mice [36]. The mechanism of these effects is unclear. GAGs bind directly to APP [37], [38], [39], [40] and may influence its function [41]. In addition, GAGs can bind to aggregated Aβ and accelerate amyloid fibril formation [42], [43].

Heparin may also influence Aβ production by disrupting APP proteolytic processing. Scholefield et al. [44] first reported that heparan sulfate and heparin can directly inhibit BACE1 activity in vitro and thereby decrease Aβ production in cell culture. Our own studies have shown that heparin binds close to the prodomain of the BACE1 zymogen (proBACE1) and that this binding stimulates proBACE1 activity [45], [46]. However, heparin can also inhibit mature BACE1 activity by binding close to the active site domain of the mature protein [46]. In contrast to the results of Scholefield et al. [44], Leveugle et al. [47] reported that heparin stimulates β-secretase cleavage of APP in a cultured cell line.

As there are conflicting reports on the effect of GAGs on APP processing and Aβ production, we have examined the effects of heparin and enoxaparin on APP processing in primary cortical cells. We used cells obtained from transgenic mice expressing human APP_695_ with the Swedish familial AD mutant (Tg2576 mouse) [48], because human APP and its fragments can be more easily detected with existing anti-human antibodies than rodent APP and Aβ.

We report that heparin and enoxaparin lower Aβ secretion from cortical cells by decreasing BACE1 and thereby inhibiting β-secretase processing of APP. This effect is not specific for amyloidogenic processing of APP, as heparin and enoxaparin also decrease the α-secretase ADAM-10 and inhibit α-secretase processing of APP.

## Results

### Effect of heparin and enoxaparin on Aβ

**Figure 1:**
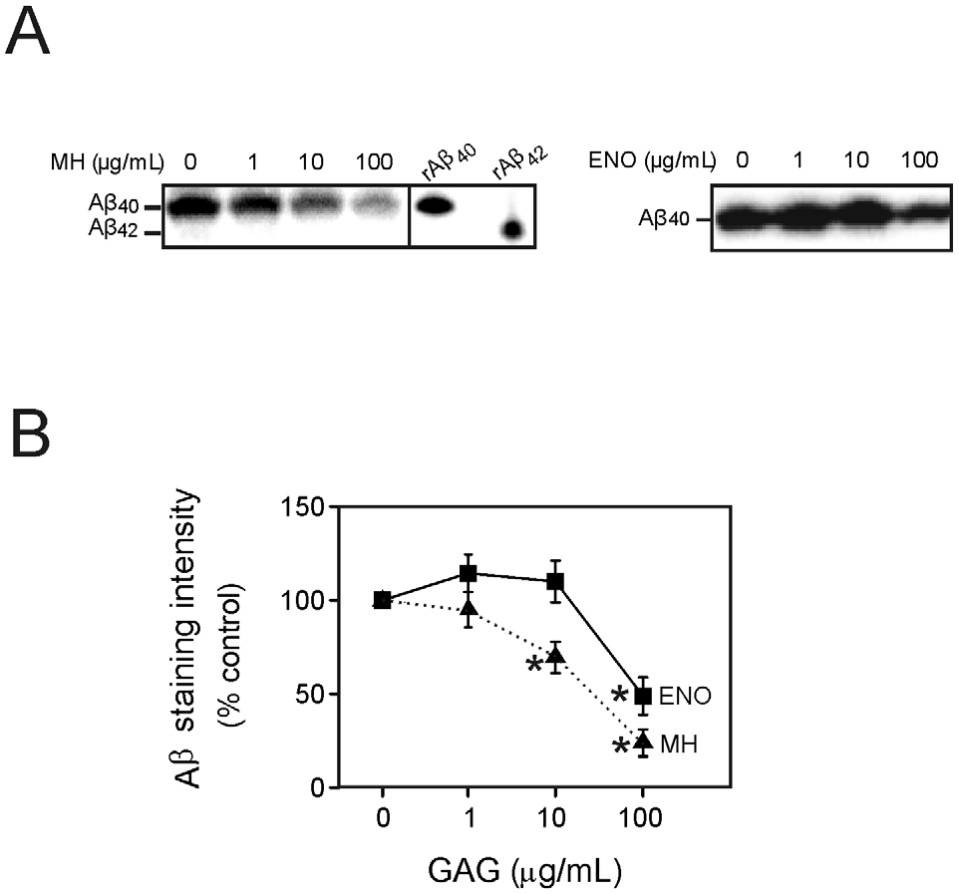
Effect of heparin (MH) and enoxaparin (ENO) on Aβ secretion from Tg2576 mouse cortical cells. Cells were treated with 0, 1, 10, or 100 µg/mL heparin or enoxaparin for 24 hours. Aβ40 and Aβ42 in the culture medium were separated on Tris-bicine-urea SDS gels and visualized by western blotting with anti-Aβ monoclonal antibody 6E10. (A) Typical western blots showing the effect of heparin and enoxaparin on Aβ40. The position of pure recombinant human Aβ40 (rAβ40) and Aβ42 (rAβ42) is also shown. (B) Quantification of Aβ40 immunoreactivity on the western blots. Asterisks show values that are significantly different from control incubations containing no GAG (*p*<0.01, n = 9).

Initially, the effect of heparin and enoxaparin on the secretion of Aβ from cortical cells was examined. Primary cortical cells from Tg2576 mice were cultured and then treated with heparin or enoxaparin for 24 hours. The cell media were harvested and Aβ was immunoprecipitated from the medium and then detected by western blotting. Although Aβ40 was easily detected in the cell culture medium, little Aβ42 was observed (Fig. 1A). Therefore, in the subsequent experiments, only the level of Aβ40 was measured. Aβ40 and Aβ42 were not detectable in the cell lysate (data not shown). Incubations with 10 µg/mL and 100 µg/mL heparin significantly lowered levels of Aβ40 in the culture medium. Enoxaparin also reduced the level of Aβ40. However, enoxaparin was less effective than heparin and only had a significant effect on Aβ40 at a concentration of 100 µg/mL (Fig. 1B).

### Characterization of APP C-terminal fragments (CTFs)

To examine the effect of heparin and enoxaparin on APP processing, we first characterized the major APP CTFs produced by the Tg2576 mouse cortical cells. The cell lysates were analyzed by western blotting using a polyclonal anti-APP C-terminal antibody.

**Figure 2:**
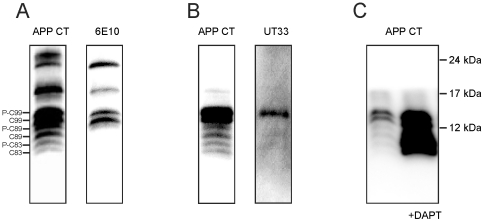
Characterization of APP C-terminal fragments in Tg2576 mouse cortical cells. (A) Cell lysates from Tg2576 primary cortical cells at 7 DIV were analysed on 16.5% Tris-tricine SDS-PAGE and immunoblotted with polyclonal anti-APP C-terminal antibody (left). Membranes were stripped and reprobed with monoclonal antibody 6E10 which recognises the N-terminal region of Aβ (right). (B) Cell lysates were immunoprecipitated using the anti-APP C-terminal antibody, and then separated on 16.5% Tris-tricine polyacrylamide gels. After blotting APP CTFs were visualized with the anti-APP C-terminal antibody (left). Blots were then stripped and reprobed with UT33 (right), an antibody which recognizes phosphorylated APP. (C) Cells were incubated in the absence (control) or presence of 0.5 µM DAPT for 24 hours. Cell lysates were resolved on 16.5% Tris-tricine polyacrylamide gels. After blotting, the APP CTFs were visualized with the anti-APP C-terminal antibody. The positions of C99, C89 and C83 as well as their phosphorylation forms (P-C99, P-C89 and P-C83) are indicated.

Analysis of the cell lysate fraction revealed six discrete protein bands migrating close to the 12-kDa molecular weight marker (Fig. 2A, left panel). These bands corresponded to phospho-C99, C99, phospho-C89, C89 (a product of cleavage adjacent to residue 11 in Aβ by BACE1), phospho-C83 and C83, based on their apparent molecular masses. To confirm their identities, the membrane was stripped and reprobed using monoclonal antibody 6E10 which recognizes C99 but not C89 and C83. As expected, we found that only the bands corresponding to phospho-C99 and C99 were stained (Fig. 2A, right panel). Next, to confirm the identification of the phospho-C99 band, the cell lysate protein was immunoprecipitated with the anti-APP C-terminal antibody, and immunoblotted using the same polyclonal anti-APP C-terminal antibody (Fig. 2B, left panel). The membrane was then stripped and restained using an anti-phospho-APP antibody UT33 which recognizes APP fragments phosphorylated at threonine 668 of the APP695 sequence [49]. Of the two 6E10-immunoreactive bands, only the upper band was immunoreactive (Fig. 2B, right panel), confirming that it was phospho-C99. Levels of phosphorylated C89 and C83 were too faint to be easily visualised using UT33. The identities of the APP CTFs were also confirmed using the γ-secretase inhibitor DAPT. DAPT has been shown previously to cause the accumulation of both β- and α-secretase-derived CTFs of APP (C99, C89 and C83) [50]. When primary cortical cells were treated with DAPT for 24 hours, there was a large increase in the levels of all protein bands around 12 kDa compared with the control (Fig. 2C), confirming the identity of these bands as APP CTFs.

### Effect of heparin and enoxaparin on levels of APP, sAPPα and CTFs

**Figure 3:**
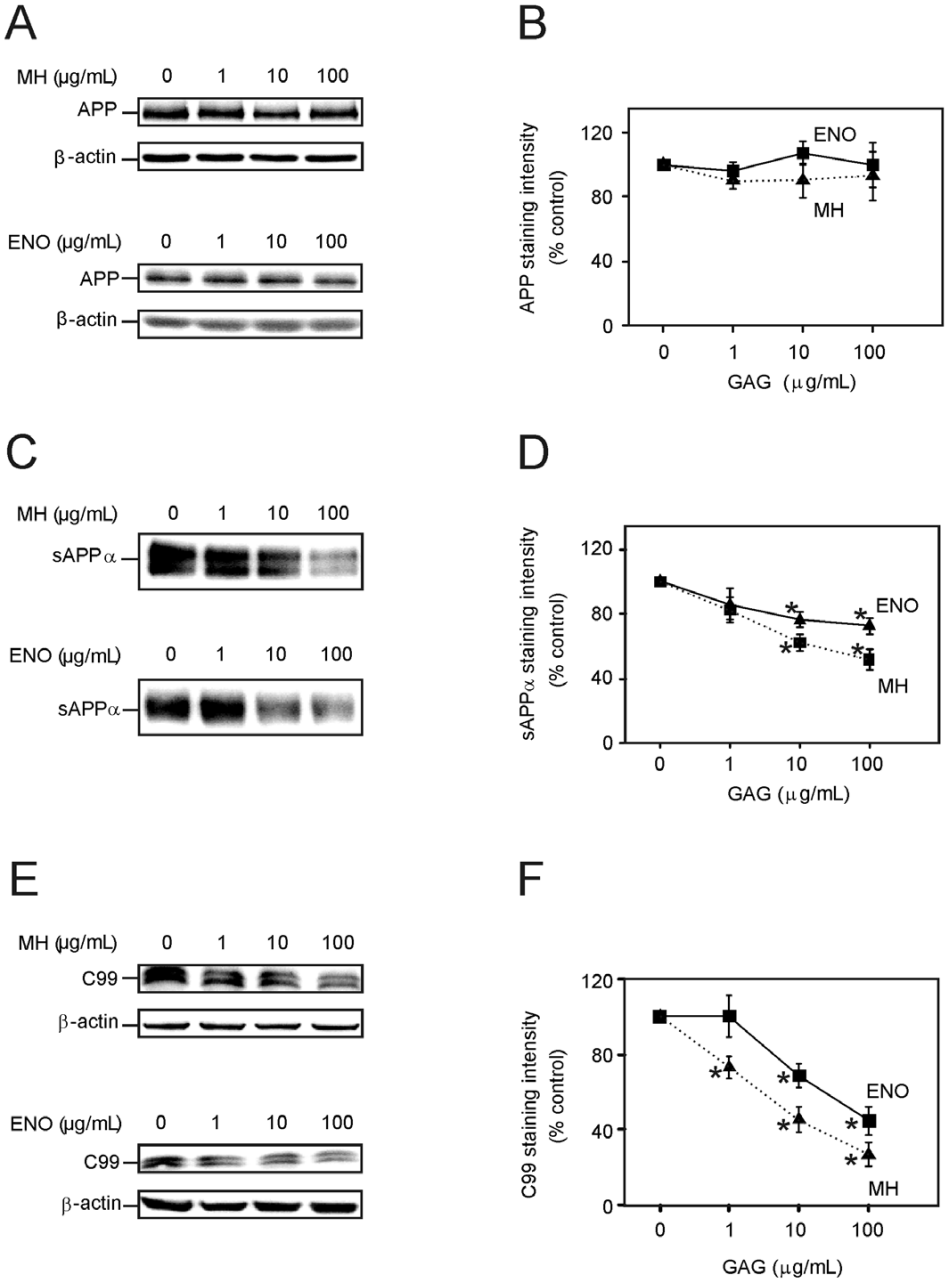
Effects of heparin (MH) and enoxaparin (ENO) on APP (A, B), sAPPα (C, D) and C99 (E, F) in Tg2576 mouse cortical cell cultures. Cells were treated with 0, 1, 10, 100 µg/mL heparin or enoxaparin for 24 hours. Figure shows typical western blots illustrating the effect of MH and ENO on APP (A), sAPPα (C) and C99 (E). β-Actin immunoreactivity is shown as a loading control. Figure also shows quantification of the level of APP (B), sAPPα (D) and C99 (F) immunoreactivity. Asterisks show values that are significantly different from controls (p<0.05, n = 12).

Next, the effect of heparin and enoxaparin on the levels of APP and the APP CTFs was examined. Heparin and enoxaparin had no significant effect on the level of full-length APP in the cell lysate (Figs. 3A and B). However, heparin decreased the amount of sAPPα secreted into the culture medium. Although 1 µg/mL heparin did not significantly decrease the level of sAPPα, higher concentrations (10 and 100 µg/mL) of heparin significantly reduced sAPPα immunoreactivity by 40% and 50%, respectively (Figs. 3C and D). Enoxaparin had a similar effect as heparin, but was less effective in its ability to reduce sAPPα. Enoxaparin (10 and 100 µg/mL) reduced the sAPPα immunoreactivity by approximately 25% and 30%, respectively.

Subsequently, the effects of heparin and enoxaparin on C99 and C83 were determined. Cell lysates were analyzed by western blotting using the anti-APP C-terminal antibody or the anti-Aβ monoclonal antibody 6E10. Although C99 was clearly visualized using this method, the level of C83 was too low to measure accurately. Therefore, only C99 was quantified. C99 and phospho-C99 were measured together as they were often poorly separated. Heparin decreased C99 in a dose-dependent manner. At concentrations between 1 and 100 µg/mL, heparin lowered C99 immunoreactivity by between 25% and 75% (Figs. 3E and F). Enoxaparin also decreased the level of C99, but it was less potent in this regard. Enoxaparin, at concentrations of 10 and 100 µg/mL, significantly reduced C99 immunoreactivity by 30% and 50% of the control value, respectively.

**Figure 4:**
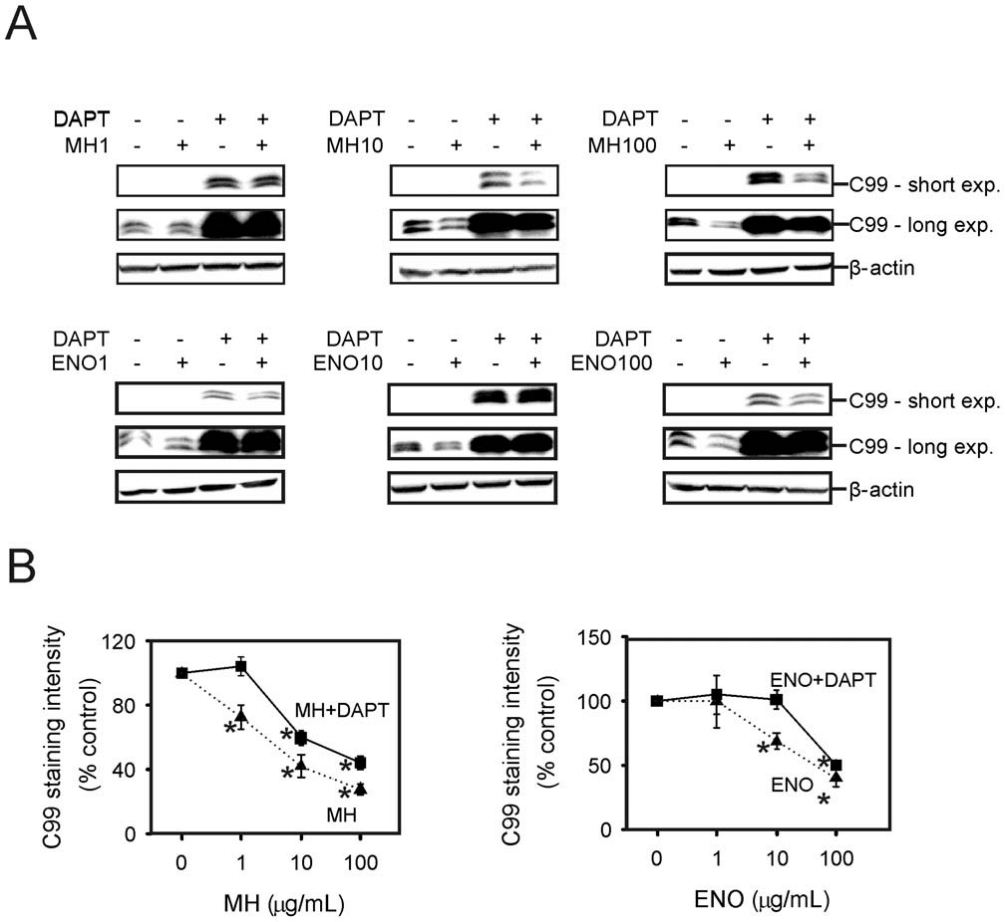
Effects of DAPT, heparin and enoxaparin on C99 levels in Tg2576 mouse cortical cells. (A) Cells were incubated in the absence (control), or presence of 0.5 µM DAPT and in the absence or presence of 1 µg/mL heparin (MH1), 10 µg/mL heparin (MH10) or 100 µg/mL heparin (MH100), 1 µg/mL enoxaparin (ENO1), 10 µg/mL enoxaparin (ENO10) or 100 µg/mL enoxaparin (ENO100) for 24 hours. The cell lysates were analysed for C99. β-Actin immunoreactivity is shown as a loading control. Chemiluminescence was detected with either short exposures (short exp.) or long exposures (long exp.) to visualise different levels of C99. (B) Quantitative analysis of C99 immunoreactivity. Asterisks show values that are significantly different from controls (p<0.01, n = 9).

**Figure 5:**
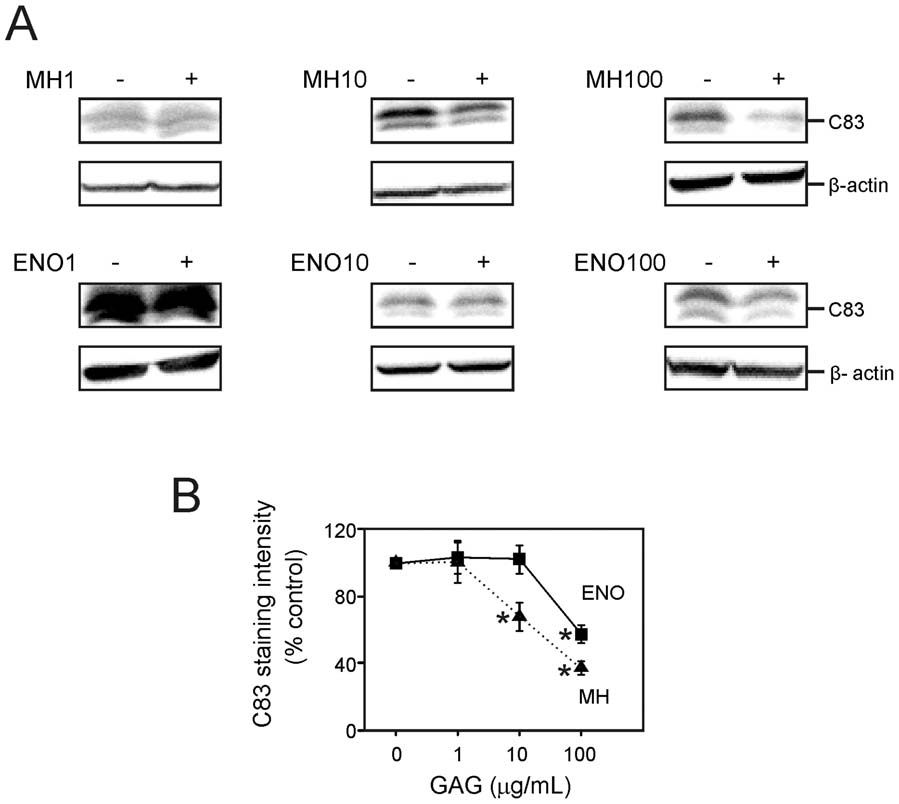
Effects of heparin and enoxaparin in the presence of 0.5 µM DAPT on C83 in Tg2576 mouse cortical cells. (A) Cells were incubated in the presence or absence of 1, 10 or 100 µg/mL heparin (MH1, MH10 or MH100, respectively) or 1, 10 or 100 µg/ml enoxaparin (ENO1, ENO10 or ENO100, respectively) for 24 hours. Cell lysates were then analysed by western blotting using polyclonal anti-APP C-terminal antibody to determine the level of C83. The level of β-actin immunoreactivity was used as a loading control. (B) Quantification of the level of C83 immunoreactivity. Asterisks show values that are significantly different from controls (*p*<0.001, n = 9).

As heparin and enoxaparin decreased levels of C99, we examined whether this decrease might be attributed to an increase in γ-secretase activity, or to a decrease in C99 production. In the presence of the γ-secretase inhibitor DAPT (0.5 µM), it was possible to accurately quantify C83 levels. Heparin (1 µg/mL) had no significant effect on the level of C99. However, 10 and 100 µg/mL heparin reduced C99 immunoreactivity by approximately 40% and 55%, respectively (Figs. 4A and B) and heparin (10 and 100 µg/mL) decreased C83 immunoreactivity by approximately 40% and 60% (Fig. 5). In the presence of DAPT, enoxaparin (1 and 10 µg/mL) did not significantly decrease C99 or C83. However, 100 µg/mL enoxaparin decreased both C99 and C83 to approximately 50% of control values (Fig. 4, Fig. 5). Taken together, the results demonstrated that the reduced secretion of Aβ was due to the decreased level of C99, and that treatment with heparin and enoxaparin also decreased α-secretase processing.

### Effect of heparin on the level of β-secretase (BACE1) and α-secretase (ADAM10 and ADAM17)

**Figure 6:**
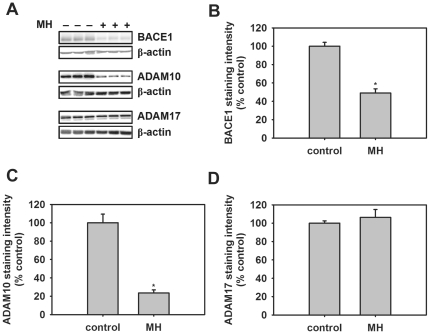
Effect of heparin (MH) on the level of BACE1, ADAM10 and ADAM17 in Tg2576 mouse cortical cell cultures. Cells were treated with 100 µg/mL MH for 24 hours. Figure shows typical western blots illustrating the effect of MH on BACE1, ADAM10 and ADAM17 (A). β-Actin immunoreactivity is shown as a loading control. Figure also shows quantification of the level of BACE1 (B), ADAM10 (C) and ADAM17 (D) immunoreactivity. Asterisks show values that are significantly different from controls (p<0.05, n = 8).

To examine whether the decrease in α- and β-secretase processing of APP was due to a reduction in the level of α- and β-secretase, primary cortical cells were treated with heparin (100 µg/ml) for 24 hr and then the cells were lysed and the level of β-secretase (BACE1) and two putative α-secretases (ADAM10 and ADAM17) were measured in the cell lysate by western blotting. Heparin (100 µg/ml) significantly decreased the level of BACE1 and ADAM10 to approximately 50% and 25% of control values (Fig. 6A–C). However, incubation with heparin did not lead to a significant change in ADAM17 levels compared with controls (Fig. 6A and D).

### Effect of heparin fragments on Aβ secretion

**Figure 7:**
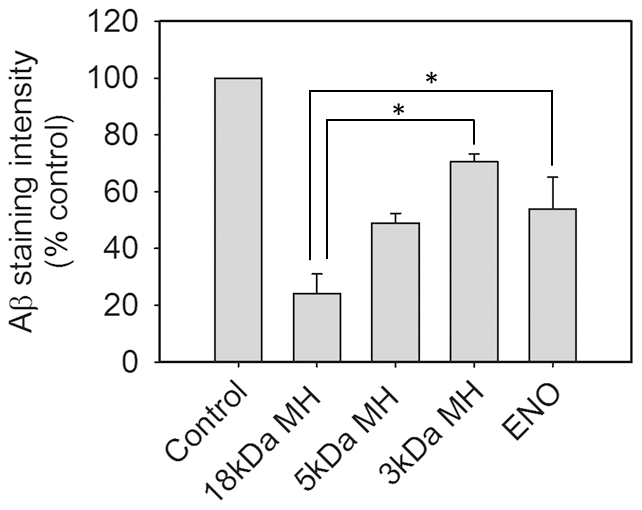
Effect of heparin fragments of different molecular weight on Aβ secretion from Tg2576 mouse cortical cells. Cells were treated with 100 µg/mL 18 kDa heparin, 5 kDa heparin, 3 kDa heparin or enoxaparin for 24 hours. Aβ40 and Aβ42 in the culture medium were visualized by western blotting using monoclonal antibody 6E10. Figure shows the quantification of Aβ40 immunoreactivity on the western blots. Asterisks show values that are significantly different from each other (*p*<0.01, n = 8).

As there were significant differences between heparin and enoxaparin in their effects on APP metabolism and Aβ production, and because enoxaparin is comprised of a mixture of small-sized heparin fragments [26], we examined the effect of heparin fragments of different molecular weight on secretion of Aβ. Primary cortical cells from Tg2576 mice were cultured and then treated with 18 kDa heparin, 5 kDa heparin, 3 kDa heparin or enoxaparin for 24 hours. The cell culture medium was harvested and Aβ was immunoprecipitated from the medium and detected by western blotting. Incubations with native 18 kDa heparin, 5 kDa heparin, 3 kDa heparin or enoxaparin significantly lowered levels of Aβ40 in the culture medium. However, 5 kDa heparin, 3 kDa heparin and enoxaparin were less effective compared with native heparin (Fig. 7).

## Discussion

In this study, the effect of heparin and enoxaparin on APP processing and Aβ production was examined in primary cortical cells obtained from APP Tg2576 mice. The study showed that while heparin and enoxaparin had no effect on the level of full-length APP, they decreased the level of C99, C83, sAPPα and secreted Aβ in a dose-dependent manner. Heparin also decreased the level of β-secretase (BACE1) and a putative α-secretase (ADAM10) but had no effect on the level of another putative α-secretase (ADAM17).

As the level of total APP was not decreased by heparin or enoxaparin, the decreased levels of C99, Aβ, sAPPα and C83 were not due to a decrease in full-length APP. Furthermore, as heparin and enoxaparin decreased the level of C83 and C99 in the presence of a γ-secretase inhibitor, this indicated that the effect of GAGs in decreasing C99 and C83 could not be due to an increase in γ-secretase activity.

We speculated that the decrease in C99, C83 and the decrease in Aβ secretion might be due to a decrease in the level of α- and β-secretases. Therefore, we measured the level of both α- and β-secretases by western blotting. Our results showed that heparin significantly decreased the level of BACE1 and ADAM10, which has been shown to be the major contributor to total α-secretase activity in many cells [19]. In contrast, the level of another putative α-secretase, ADAM17 was not changed in the presence of heparin. Therefore, the results demonstrate that the GAG-induced decrease in Aβ secretion and the reduction in C99 were most likely due to a decrease in BACE1 levels within the cortical cells.

The mechanism by which GAGs lower levels of BACE1 and ADAM10 is unclear. As BACE1 may bind to endogenous proteoglycans and because this interaction may be important for the trafficking [44] or processing of BACE1 [45], it is possible that heparin and enoxaparin may disrupt the normal trafficking or processing of the enzyme. However, it is unclear whether such a mechanism can also explain the effect of heparin on ADAM10 levels. Furthermore, it was interesting to note that the structurally related enzyme ADAM17 was not affected by heparin. To date, there have been no reports that ADAM10 can interact with GAGs or proteoglycans, although this possibility cannot be ruled out. GAGs could conceivably exert a less direct effect on BACE1 and ADAM10, possibly by acting on a specific secretory pathway. In this regard, it is interesting to note that there are differences in the roles of ADAM10 and ADAM17 in membrane protein shedding [51]. ADAM17 can be stimulated by protein kinase C and may be part of a regulated secretion pathway [52], whereas ADAM10 may be associated with a constitutive secretion pathway [53]. APP can be processed via both pathways [15], [16], [54]. Thus it is tempting to speculate that GAGs may selectively alter the level of enzymes in the constitutive secretion pathway, rather than the regulated pathway, thus accounting for the selective decrease in ADAM10 but not in ADAM17.

We found that the smaller sized enoxaparin was less efficient in its ability to inhibit APP processing to Aβ than native full-length heparin. To examine the reason for this difference, we compared 18 kDa native heparin and enoxaparin with heparins of defined molecular weight. We found that low molecular weight (3 kDa and 5 kDa) heparins were less effective in inhibiting the secretion of Aβ than native heparin (Fig. 7). Enoxaparin possesses an average molecular weight of approximately 4.5 kDa and its efficacy for inhibiting Aβ secretion was similar to that of 5 kDa heparin. Therefore, this result suggests that the differences between heparin and enoxaparin are probably due to differences in molecular weight.

In a previous preliminary study, we reported that heparin could decrease the level of Aβ and sAPPα in cortical cells [55]. However, in that study, we did not observe any effect of heparin on C99 or C83. Our failure to observe an effect on C99 and C83 previously was possibly due to the fact that the number of replicates in the earlier experiments was too low to observe significant differences in C99 and C83 levels. In a previous study, Scholefield et al. [44] reported that heparin and heparin analogues inhibit Aβ and sAPPβ generation in APP-expressing cells, and they drew the conclusion that GAGs may bind to BACE1 and inhibit BACE1 cleavage activity. Our own studies have shown that at low concentrations (e.g. 1 µg/ml) GAGs stimulate the activity of the zymogen pro-BACE1 whereas at higher concentrations (e.g. 100 µg/ml) they inhibit the activity of the mature enzyme [45]. This biphasic effect of GAGs is most simply explained by the presence of a single heparin-binding site that lies adjacent to the both the pro sequence and active site regions [46]. Low concentrations of GAGs can alter the conformation of the prodomain, but higher concentrations are needed to block the active site [46].

In contrast to our study, Leveugle et al. [47], using SH-SY5Y neuroblastoma cells overexpressing APP with the Swedish mutation, reported that GAGs can increase APP secretion and processing through the β-secretase pathway. The difference in the effect of GAGs between the study of Leveugle et al. [47] and our own data is unclear, but it may possibly be explained by the fact that APP processing occurs differently in neuroblastoma cells overexpressing APP compared with primary cortical cells. This possibility emphasizes the need to use primary cells in culture, rather than cell lines for these types of studies.

Interestingly, we found that, in the absence of the γ-secretase inhibitor DAPT, C99 was more abundant than C83. This finding contrasts with reports from many studies using APP-transfected cells that C83 is more abundant than C99 [56], [57], in keeping with the concept that the α-secretase pathway is a predominant route of APP processing than the β-secretase pathway. However, the observation that C99 was present in higher abundance than C83 is consistent with some previous studies in both APP23 and Tg2576 mice [58], [59], [60]. One possible explanation for the high level of C99 in the Tg2576 cells is that the transgene possesses the human Swedish NL double mutation at the beginning of the Aβ encoding region. This mutation increases the susceptibility of APP for cleavage by BACE1 [61], [62]. Another reason why the level of C99 may have been higher than C83 is that the relative rate of cleavage of C99 and C83 by γ-secretase may be different. The level of C99 and C83 depends, in part, on the relative rates of α- and β-secretase cleavage of APP [15]. However, it may also depend upon the capacity of the cells for γ-secretase cleavage. It has been reported that the γ-secretase degrades C83 much quicker than C99 [60]. Under conditions in which the γ-secretase is not saturated with its substrate, the relative rates of cleavage of C83 and C99 by γ-secretase may greatly influence the level of total C83 or C99. However, under conditions of very high APP overexpression, or under conditions where γ-secretase is almost completely inhibited, the γ-secretase would be saturated and the relative proportions of C99 and C83 would be dependent solely upon the relative rates of β- and α-secretase cleavage, respectively. In support of this idea, we found that in the presence of the γ-secretase inhibitor DAPT, the proportion of C83 relative to C99 increased greatly.

In summary, our experiments demonstrate that GAGs alter APP metabolism and decrease secretion of Aβ. Low molecular weight heparins have also been shown to cross the blood-brain barrier [28], [29], to arrest amyloid-induced inflammation [63], [64], to decrease Aβ aggregation [64] and to lower the Aβ generation and improve cognition in AD transgenic mice [32], [36]. Therefore, our data provide support for the view that heparin analogues may have value for the treatment of AD. However, it is worth considering the possibility that heparin analogues may have toxic side effects which lower their value as therapeutic agents. Our study found that GAGs inhibited α-secretase processing of APP, and this effect was associated with a decrease in the level of ADAM10. A number of studies have suggested that sAPPα may have important trophic functions [65]. Thus inhibition of sAPPα production could produce adverse effects in vivo. Such a possibility needs to be considered when examining the potential of heparin analogues for the treatment of AD.

## Materials and Methods

### Materials

Porcine mucosal heparin, 5 kDa heparin, 3 kDa heparin, monoclonal anti-β-actin antibody, rabbit anti-BACE1 (EE-17) antibody, polyclonal anti-APP C-terminal antibody (APP-CT) and N-[N-(3,5-difluorophenacetyl)-L-alanyl]-S-phenylglycine *t*-butyl ester (DAPT) were purchased from Sigma-Aldrich Pty. Ltd. (Sydney, Australia). Rabbit anti-ADAM10 (ab1997) and rabbit anti-ADAM17 (ab2051) were purchased from Sapphire Bioscience Pty. Ltd. (Waterloo, Australia). Monoclonal anti-Aβ antibody 6E10 was from Covance Pty. Ltd. (North Ryde, Australia). Neurobasal medium and B27 supplement were purchased from Invitrogen (Mulgrave, Australia). Mouse and rabbit HRP-conjugated secondary antibodies were purchased from DAKO (Campbellfield, Australia). Enoxaparin sodium (Clexane®) was from Sanofi-Aventis (Macquarie Park, Australia). Protein G Sepharose and complete mini protease inhibitor cocktail tablet were purchased from Roche Diagnostics (Castle Hill, Australia). A polyclonal anti-phosphorylated APP antibody UT33 was prepared as previously described [49].

### Cell culture

Cortical cells were prepared from newborn (P_0_) Tg2576 mouse. Cerebral cortices were dissected and incubated in Ca^2+^- and Mg^2+^-free Hank's balanced salts (HBSS) containing 0.25% (w/v) papain and 0.06% (w/v) deoxyribonuclease I (DNase I) for 30 min at 37°C, followed by three washes with neurobasal medium. Cells were then separated by gentle mechanical dissociation and 3×10^5^ cells were plated onto poly-D-lysine-coated 12-well culture plates, and maintained in 1.2 mL complete neurobasal medium containing 2% B27 supplement, 1 mM glutamine, and 1% penicillin/streptomycin (10,000 units of penicillin and 10,000 µg of streptomycin stock) in an atmosphere containing 5% CO_2_ at 37°C. After 3 days in vitro (DIV), half of the culture medium was replaced with fresh complete neurobasal medium. All experiments were performed at 7 DIV cultures. Primary cortical cells were incubated with GAGs and inhibitors for 24 hours prior to sodium dodecylsulfate polyacrylamide gel electrophoresis (SDS-PAGE).

### SDS-PAGE and western blotting

After treatment with GAGs or drugs, the medium was removed from cells for determination of Aβ and sAPPα. The cells were incubated with cold RIPA buffer (150 mM NaCl, 50 mM Tris, 0.5% w/v Na-deoxycholate, 1% v/v Nonidet P-40, 0.1% SDS, pH 7.4) containing protease inhibitor cocktail on ice for 10 min and the cell lysates were then harvested for determination of C99, C83,BACE1, ADAM10, ADAM17 and full-length APP.

The amount of Aβ40 or Aβ42 secreted into cell medium was determined on 15% Tris-bicine-urea SDS-PAGE gels as described previously [66]. The cell medium was collected and cell debris was removed by centrifugation at 500 g for 5 min. The supernatant fractions were then transferred to 1.5 mL Eppendorf tubes. Monoclonal antibody 6E10 (1∶1667 v/v) was added and the mixture was incubated overnight at 4°C. Protein G agarose (20 µL hydrated gel/mL medium) was then added and incubated for a further 3 hours at 4°C. The beads were washed 3 times in 1 mL cold phosphate-buffered saline (PBS), and then 30 µL of sample buffer was added to each pellet. After gentle mixing, the slurry was heated for 5 min at 95°C. The sample was then centrifuged for 10 min at 15,000 rpm and 30 µL supernatant fraction was loaded on 15% Tris-bicine-urea gels. After electrophoresis, proteins were transferred onto a polyvinylidene difluoride (PVDF) membrane and stained with monoclonal antibody 6E10 (1∶2,000 dilution) for Aβ.

For the determination of sAPPα, BACE1, ADAM10, ADAM17and full-length APP, cell medium (the loading volumes were normalized from protein concentration of cell lysates) or 8 µg protein cell lysate were applied to 8% Tris-glycine SDS-PAGE gels and subjected to western blotting using the anti-Aβ monoclonal antibody 6E10 (1∶2,000 dilution), anti-ADAM10 polyclonal antibody (1∶1,000 dilution), anti-BACE1 polyclonal antibody (1∶1,000 dilution), anti-ADAM17 polyclonal antibody (1∶1,000 dilution) or an anti-β-actin monoclonal antibody (1∶16,000 dilution). The protein concentration was measured using the Bio-Rad DC protein assay kit (Bio-Rad Laboratories Pty. Ltd., Gladesville, Australia) with bovine serum albumin as standard.

To determine the level of C99 and C83 in cells, cell lysates containing 12 µg of protein were applied to 16.5% Tris-tricine SDS-PAGE gel. After electrophoretic transfer of proteins onto a PVDF membrane, the membrane was cut into two pieces at the position of the 24 kDa molecular weight marker. The piece of PVDF membrane containing proteins from the upper region of the gel which migrated above an apparent molecular mass of 24 kDa was stained using a monoclonal antibody directed against β-actin (1∶16,000 dilution), and the piece containing proteins from the lower region of the gel which migrated below 24 kDa was stained for C99 and C83 using a polyclonal anti-APP C-terminal antibody (1∶2,000 dilution), which was raised against residues 676–695 of the APP695 sequence.

For all western blotting, the bound primary antibody was detected using either a polyclonal goat anti-mouse or an anti-rabbit immunoglobulin conjugated to horseradish peroxidase (HRP) (1∶6,000 dilution) and Immobilon™ Western chemiluminescent HRP substrate from Millipore Pty. Ltd. (North Ryde, Australia). Chemiluminescence was detected using a Chemi-Smart 5000 gel documentation system (Vilber Lourmat, Torcy, France). Images were taken and the density of staining was quantified using Image J software (RSB; NIH, http://rsbweb.nih.gov/ij/index.html). The ratio of immunoreactivity for each protein to the β-actin immunoreactivity was determined and then each ratio was used to calculate a percentage relative to mean values for control incubations lacking GAG. All experiments were performed at least six times and statistical tests were performed using SigmaPlot software (10.0v; Systat Software, Inc., San Jose, CA, USA). Statistical comparisons were made using one-way analysis of variance and Student's t tests. Values of *p*<0.05 were considered statistically significant.
